# Peripheral nerve block with a high concentration of tetracaine dissolved in bupivacaine for intractable post-herpetic itch: a case report

**DOI:** 10.1186/s40981-016-0069-y

**Published:** 2016-12-05

**Authors:** Daiki Yamanaka, Takashi Kawano, Marie Shigematsu-Locatelli, Atsushi Nishigaki, Sonoe Kitamura, Bun Aoyama, Hiroki Tateiwa, Noriko Kitaoka, Masataka Yokoyama

**Affiliations:** Department of Anesthesiology and Intensive Care Medicine, Kochi Medical School, Kohasu, Oko-cho, Nankoku, Kochi 783-8505 Japan

**Keywords:** Post-herpetic itch, Peripheral nerve block, Tetracaine

## Abstract

**Background:**

Post-herpetic itch (PHI) is a neuropathic itch syndrome following herpes zoster. It has been reported that PHI is occasionally sufficiently severe to compromise patients’ quality of life and frequently refractory to treatment. Here, we present a case of severe chronic PHI successfully treated with supraorbital nerve block using a high concentration of tetracaine dissolved in bupivacaine.

**Case presentation:**

An 82-year-old man presented with severe chronic itching in the ophthalmic branch of the left trigeminal nerve dermatome, following acute herpes zoster. The patient’s itching was unresponsive to usual medical treatments for PHI including antiepileptic drugs, topical capsaicin cream, and supraorbital nerve radiofrequency thermo-coagulation. Topical lidocaine cream could relieve the itching, but could not provide long-term relief of itching and thus failed to achieve a satisfactory result. After these conventional treatments, left supraorbital nerve block using 4% tetracaine dissolved with 0.5% bupivacaine was conducted. Afterwards, the patient experienced long-lasting resolution of the itching with improvement of sleep disturbance. A transient, mild edema of the eyelids occurred, but there were no other complications.

**Conclusions:**

Peripheral nerve block using 4% tetracaine dissolved with 0.5% bupivacaine was beneficial in relieving PHI in the ophthalmic division of the trigeminal nerve.

## Background

Pain persisting after acute herpes zoster, termed post-herpetic neuralgia (PHN), is one of the most common causes of neuropathic pain [1, 2]. The symptoms of PHN can potentially last for years, are often difficult to treat, and have been shown to adversely affect patients’ quality of life. In addition to the conventional pain symptoms, approximately half of the patients with PHN may experience itching, which is occasionally severe [3, 4]. This is known as post-herpetic itch (PHI). PHI occurs in the herpes zoster-affected dermatome, and is especially common in the cases of herpes zoster involving the head or neck. Based on both literature and clinical experience, severe PHI may be even more disabling and harder to treat than the pain symptoms associated with PHN [5]. As PHI is induced without the involvement of histamine, antihistamines are generally ineffective in treatment. There is currently no established approach for the management of PHI, and the efficacy and safety of therapeutic modalities are uncertain. Here, we describe a case of intractable PHI, which was successfully managed with peripheral nerve block by a high concentration of local anesthetics.

## Case presentation

An 82-year-old, 152 cm, 68 kg male patient had decompression surgery for cervical ossification of the posterior longitudinal ligament (C4/5 to C5/6) at a local hospital. One week after the surgery, a burning pain followed by eruption of a rash appeared in the left aspect of the head extending to the side of the nose, upper eyelid, frontal, parietal, and temporal area. Although the patient did not exhibit any eye symptoms, he reported severe itching in the affected areas. A clinical diagnosis of acute herpes zoster on the left ophthalmic branch of the trigeminal nerve was confirmed by a dermatologist, and the patient was treated with acyclovir (400 mg, five times a day) and non-steroidal anti-inflammatory drugs for 1 week. Oral tramadol 25 mg four times daily was also prescribed. The rash was resolved within 2 weeks, and his own numerical rating scale pain score reduced from 6/10 to 2/10 (0/10 = no pain and 10/10 maximum pain). However, supraorbital itching remained severe. Topical lidocaine cream (10%) could relieve the itching, but its efficiency was temporary, a few hours at most, and it failed to obtain enough of an improvement in the patent’s reported quality of life. The patient was also treated with 0.075% capsaicin cream for 5 weeks, but no significant change was seen. Pregabalin (75 mg twice daily) was started, but no improvement was noted and the patient reported experiencing an intolerable side effect of dizziness. Then, pregabalin was replaced by amitriptyline (starting 10 mg daily for 1 week, and 30 mg daily for 2 weeks), clonazepam (1.5 mg daily for 1 week), or carbamazepine (200 mg daily for 2 weeks); however, itching intensity and characteristics did not change. The patient was then referred for consultation to our department approximately 6 months after the onset of symptoms.

On examination, the severe itching in the dermatomal distribution of the left ophthalmic division of the trigeminal nerve occurred throughout the day and worsened at night, causing sleep loss and considerably affecting the patient’s quality of life. An acquired herpetic scar with self-injury from scratching was observed in the distribution of the left supraorbital nerve. The intolerable lancinating itching can be triggered by touching the affected areas. On the other hand, the patient had no sensory signs of allodynia/hyperalgesia, and his PHN-associated pain was mild (1/10–3/10). Vision, corneal reflex, and laboratory tests were normal. He had no history of any other significant past medical conditions.

**Fig. 1:**
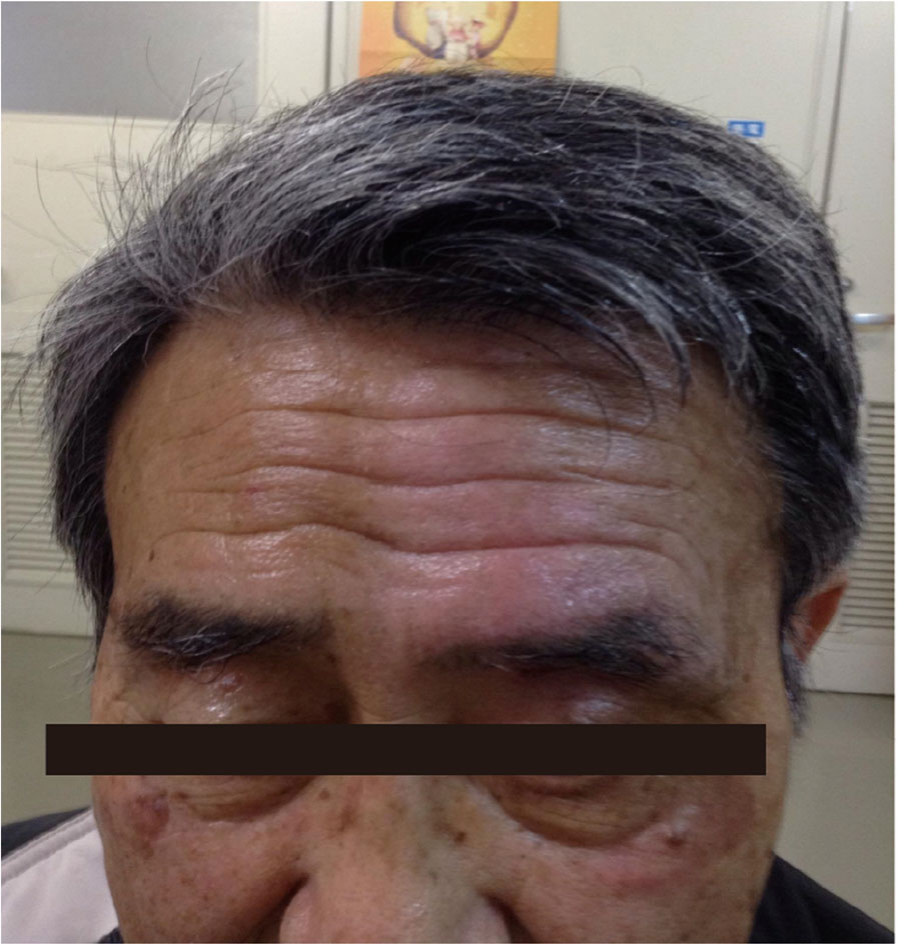
Photograph of the patient taken at 1 week after the supraorbital nerve block using 4% tetracaine dissolved with 0.5% bupivacaine. Mild edema around his left eyelids was observed

As the severe itching remained refractory despite any pharmacological treatments, we performed peripheral branch neurolysis therapy for this patient. A diagnostic left supraorbital nerve block was initially performed using 0.5 ml of 1% lidocaine. This resulted in short-term, but complete relief of the itching. One week after this block, radiofrequency thermo-coagulation (RT) was performed in the left supraorbital nerve. The patient was placed in the supine position. The supraorbital notch was easily palpated in the medial aspect of the supraorbital ridge. A radiofrequency needle insulated with a 4-mm active tip (22G) was slowly introduced, and the tip was slipped into the notch. The supraorbital nerve was confirmed using sensory stimulation at 50 Hz and 0.5 V. After local anesthesia, RT with tip temperature of 90 °C and duration of 90 s was performed. The procedure was uneventful and he was discharged home. However, the patient returned 1 week later reporting of continued itching with recurrence of the baseline. We considered that the treatment was unsuccessful due to the limited RT lesion area, and thus did not ablate all itch-mediating fibers. Therefore, we recommended to repeat the procedure with higher intensity RT. However, the patient refused this procedure expressing his concerns about potential side effects that were explained during informed consent. Instead, the patient agreed to another treatment, i.e., a left supraorbital nerve block using a high concentration of local anesthetics. During the procedure, the patient was placed in the supine position. A 25-gauge needle was inserted at the level of the supraorbital notch, and then 0.2 ml of 4% tetracaine dissolved in 0.5% bupivacaine (20 mg tetracaine was dissolved in 0.5 ml of 0.5% bupivacaine) was slowly injected. One week after the block, itching was relieved almost completely, and neither allodynia nor paresthesia was observed in the distribution of the left supraorbital nerve. The patient developed edema of the eyelids during first 2 weeks (disappeared within 3 weeks), which was an acceptable side effect (Fig. 1). No other complications were noted. The improvement of itching and related sleep disturbances has continued for 6 months. Approximately 8 months later, the patient received the same block and a similar good outcome was obtained.

### Discussion

Although both intact pain and itch sensations are essential in everyday life, chronic hyper-sensation can be a debilitating disease state without any useful value. PHN is one of the most severe health problems that can cause chronic pain and itch simultaneously or alone [1, 2]. In this report, we describe a patient with severe PHI in the ophthalmic division of the trigeminal nerve without significant pain. Itching worsened, especially at night, and interfered with sleep, resulting in a significant reduction of the patients’ quality of life. The symptoms of itching were refractory to conventional medications, but were resolved following supraorbital nerve block using 4% tetracaine dissolved with 0.5% bupivacaine for a duration of 6 months with minimum side effects.

The exact mechanisms underlying neuropathic itch including PHI are largely unknown. Current research has focused on several factors that may contribute to the development of PHI, including selective preservation of peripheral itch-specific C-fibers, imbalance itch signaling between excitation and inhibition within the dorsal horn, and/or hyperactivity of central itch-specific neurons [3]. In addition, there may be common pathogenesis between neuropathic itching and pain such as peripheral and central sensitization [6]. Therefore, although no specific trials of medications for PHI have been conducted, one could expect that the pharmacological therapies for neuropathic pain may be effective for neuropathic itching as well. Indeed, there are case reports that indicated the anticonvulsants pregabalin and gabapentin, well-accepted drugs for neuropathic pain, to be effective against neuropathic itch [7–10]. Both of the drugs can bind to the α2δ subunit of voltage-gated calcium channels and reduce excitatory neurotransmitters, which may account for the analgesic and anti-pruritic effects. In contrast, much anecdotal evidence has documented that medications that alleviate neuropathic pain are not as effective in relieving neuropathic itch [3, 11, 12]. Consistently, the symptoms of PHI in our patient were refractory to pregabalin and other drugs available for neuropathic pain. Further prospective clinical trials will be needed to investigate this discrepancy.

The topical administration of lidocaine, available as a form of cream, gel, or patch, can reduce the small afferent sensory signals at the nerve endings in the skin by blocking voltage-gated sodium channels. Therefore, it has long been used to treat burning pain or itching [13, 14]. For pain management, there is evidence that the 5% transdermal lidocaine patch is effective for allodynia associated with PHN [15]. The reported side effects are only mild skin reactions. Iseki and colleagues also demonstrated that topical application of 10% lidocaine cream is a safe and effective treatment for pain associated with sub-acute herpes and PHN [16]. Regarding the management of itching, there are case reports of effective management of patients with notalgia paresthetica, pruritus ani, and post-burn pruritus using topical lidocaine [14]. Similarly, the chronic itching in our patient was successfully reduced with 10% lidocaine cream, but it cannot provide long-term relief of itching, i.e., only few hours of effectiveness. As the patient had to apply the cream several times, especially at night, to control the itching, this treatment failed to obtain enough of an improvement of his sleep disturbance.

Interventional therapies may be recommended to patients with peripheral neuropathic syndrome refractory to conventional medical management, like our patient. Peripheral nerve block with local anesthetics may contribute to relieve the symptoms, but it usually does not provide long-term effects. Therefore, one generically applicable interventional technique is ablative peripheral nerve block due to its persistence, as well as selectivity, minimal invasiveness, and convenience. Among the procedures, the peripheral branch RT is reported to be a highly effective approach for treating trigeminal neuralgia [17, 18]. During RT, the radiofrequency current is emitted to heat up the tip of the needle placed in the vicinity of a target nerve and a lesion is formed, thereby interrupting pain signals. However, in our case, despite the positive response to the diagnostic supraorbital nerve block, RT was ineffective for relieving the itch. As the area of the RT lesion may be limited compared with the infiltration area of local anesthetics, RT could not ablate all of the itch-mediating fibers and failed to achieve symptom control. The increase in tip temperature, lesion duration, needle diameter, and length of the active tip is known to contribute to enlarging the RT lesion [17–19]. However, albeit less common, it may also be associated with complications such as anesthesia dolorosa, motor paresis, and miscellaneous eye problems [19, 20].

A peripheral neurolytic nerve block has also been shown to provide long-term pain relief [21–23]. In particular, patients who have positively responded to a diagnostic block may be suitable candidates for this intervention. An alcohol (50–100%) or phenol (5–15) is currently used as neurotoxic chemical substances. These agents can produce nerve destruction by protein denaturation and extraction of membrane phospholipids to disrupt pain signals [23]. However, a high rate of substance-related side effects, e.g., hypoesthesia, paresthesia, tissue narcosis, and eye complications, has been reported after the block [21–24].

Alternatively, a high concentration of local anesthetics may also be used as neurolytic agents. Preclinical studies demonstrated the dose-dependent local anesthetic-induced neurotoxicity [25]; specifically, tetracaine could produce neurotoxicity at concentrations of more than 1% [26], which may explain its long-term analgesic effects. Mechanisms for local anesthetic neurotoxicity remain speculative, but previous studies have demonstrated that local anesthetic-induced neurotoxicity may be associated with altered perineural permeability, endoneurial oedema, and Wallerian degeneration [27, 28]. Clinically, previous reports showed that the trigeminal nerve block using 4% tetracaine dissolved with 0.5% bupivacaine could provide prolonged analgesia (several months) in patients with trigeminal neuralgia, without long-lasting complications or sensory disturbance [29, 30]. These findings imply that trigeminal nerve block using high concentration of tetracaine is reversible and safer than that using the neurotoxic chemical substances. The present report further demonstrates that the trigeminal nerve block using 4% tetracaine dissolved with 0.5% bupivacaine was also effective for long-term treatment of neuropathic itching. Due to its safety, this procedure could be repeated in the same patient if required. These favorable benefits may facilitate the application of this technique for chronic severe refractory PHI. Further clinical studies, including case studies and treatment experience, are necessary to confirm this point of view.

## Conclusions

This case report demonstrates that the supraorbital nerve block using 4% tetracaine dissolved with 0.5% bupivacaine may be a promising treatment option for chronic PHI refractory to conventional medical therapy.

## Abbreviations

PHI: Post-herpetic itch
PHN: Post-herpetic neuralgia
RT: Radiofrequency thermo-coagulation
